# An Influence Maximization Algorithm for Dynamic Social Networks Based on Effective Links

**DOI:** 10.3390/e24070904

**Published:** 2022-06-30

**Authors:** Baojun Fu, Jianpei Zhang, Hongna Bai, Yuting Yang, Yu He

**Affiliations:** 1 College of Computer Science and Technology, Harbin Engineering University, Harbin 150001, China; 2 College of Computer Science and Information Engineering, Harbin Normal University, Harbin 150025, China; hrbnub@gmail.com (H.B.); savannay998@gmail.com (Y.Y.); heyu-apple@hrbnu.edu.cn (Y.H.)

**Keywords:** influence maximization, dynamic social networks, effective link, independent cascade model

## Abstract

The connection between users in social networks can be maintained for a certain period of time, and the static network structure formed provides the basic conditions for various kinds of research, especially for discovering customer groups that can generate great influence, which is important for product promotion, epidemic prevention and control, and public opinion supervision, etc. However, the computational process of influence maximization ignores the timeliness of interaction behaviors among users, the screened target users cannot diffuse information well, and the time complexity of relying on greedy rules to handle the influence maximization problem is high. Therefore, this paper analyzes the influence of the interaction between nodes in dynamic social networks on information dissemination, extends the classical independent cascade model to a dynamic social network dissemination model based on effective links, and proposes a two-stage influence maximization solution algorithm (Outdegree Effective Link—OEL) based on node degree and effective links to enhance the efficiency of problem solving. In order to verify the effectiveness of the algorithm, five typical influence maximization methods are compared and analyzed on four real data sets. The results show that the OEL algorithm has good performance in propagation range and running time.

## 1. Introduction

With the rapid development of social networks, more and more people use Weibo, WeChat, Twitter, Facebook and other social software to carry out information exchange, product promotion [1], public opinion propaganda [2] and other activities, which bring great convenience to people’s productive life. Viral marketing [3], which is carried out especially in social networks using the word-of-mouth effect, can quickly enable a large number of customers to learn about and buy products through social connections, maximizing the benefits for businesses. The core problem of this process is to find the target group with good communication characteristics, and with the help of the relationship network formed among users, more and more users receive the product and achieve the maximum promotion of the product, and this process is the problem of maximizing influence.

Currently, the main approach to influence maximization problem research is to abstract social networks as static structures, ignoring the fact that the interactions between users change over time. In particular, in some special network structures, such as telephone communication, email transmission, and transportation networks, users do not maintain connections with each other all the time, but only connect at a certain moment or time period, which fully demonstrates that the connection between users in social networks is time sensitive. As a result, the traditional research methods cannot be applied to influence maximization in dynamic social networks, and the research on such problems will face the following challenges.

- The traditional propagation models mainly focus on the study of information diffusion in static networks, which cannot reflect the changes of node interaction in dynamic social networks well;
- The spread of influence should conform to the timeliness of the connection between users in social networks, and the diffusion mechanism can only play a role in the effective stage;
- The weight indicator to measure the mutual influence among users in social networks should be combined with the time factor.

In view of the above problems, the main contributions of this paper include the following aspects.

1. The functions of out-degree neighbors and in-degree neighbors in social networks are refined, and the influence probability between users is reset according to the topological relationship between users’ direct neighbors and indirect neighbors;
2. The traditional independent cascade model is improved by taking dynamic social networks as the research object, and the time-based propagation model is proposed by combining the effective number of connections between users;
3. A two-stage influence maximization algorithm, Outdegree with Effective Link (OEL), is proposed to solve the problem of selecting seed nodes on dynamic social networks by combining submodular properties;
4. The effectiveness of the OEL algorithm is verified by experimental comparison.

The rest of the article is organized as follows: In Section 2, we discuss some of the work related to maximizing influence. In Section 3, related concepts, propagation models and setting methods of influence probability among users of social networks are introduced around dynamic social networks. In Section 4, we first propose a time-series based propagation model to measure the process of information transmission in dynamic social networks. Secondly, a two-stage dynamic social network influence maximization algorithm, OEL, is developed to achieve target seed node screening. In Section 5, we use the proposed algorithm on real data sets to realize the selection of seed nodes in the influence maximization problem. Through comparative analysis with other methods, we demonstrate the advantages of the algorithm in the propagation range and time efficiency. Finally, in Section 6, we summarize the work and propose the research plan for the next step.

## 2. Related Works

Domingos and Richardson [4] analyzed the characteristics of information transmission in social networks for the first time and proposed the issue of influence maximization. Kempe [5] defined the influence maximization problem as a discrete optimization problem and proved that the influence maximization problem is an NP-hard problem in the Independent Cascade (IC) model and Linear Threshold (LT) model. A greedy algorithm is proposed to solve this kind of problem, and an approximate optimal solution of 1-1/e-ε can be obtained. Because a greedy algorithm needs Monte Carlo simulation for many times, the algorithm runs for a long time. In order to improve the operating efficiency of the algorithm, Leskovec et al. [6] proposed the CELF (cost-effective lazy-forward) algorithm to reduce the number of Monte Carlo simulations using the submodular property, and the experimental results proved that the speed was nearly 700 times faster than the greedy algorithm. In addition, many researchers have proposed heuristic algorithms [7,8,9,10,11], which further improve the efficiency of the algorithm. The two representative approaches are the degree centrality algorithm [12] and the betweenness centrality algorithm [13]. These two algorithms analyze the importance of nodes in social networks from the global scope and the local scope, respectively. The degree centrality algorithm ignores the difference of directly adjacent nodes and only pays attention to their number, which leads to the inability to distinguish important nodes well, such as nodes with the same degree, and it is more important to include nodes with greater propagation ability in the neighbors. Because the degree attribute of the nodes in the social network exhibits the characteristics of power-law distribution, the nodes with a high number of heights are distributed in a concentrated manner, which leads to the centrality algorithm not being able to deal with the overlapping influence problem well. The betweenness centrality algorithm analyzes the importance of nodes in the process of information dissemination from the global scope according to the number of shortest paths passing through nodes in the network. However, the algorithm restricts the information to be propagated through the shortest path, which does not conform to the law of social network information propagation, and the calculation process requires knowledge of the overall network structure information, resulting in low computational efficiency and is unsuitable for large-scale social networks. In order to avoid the drawbacks of the above methods, many heuristic methods have been proposed from different perspectives, including a heuristic algorithm based on k-kernel proposed by Cao Jiuxin et al. [14]. It improves the operation efficiency and covers many important nodes, but the operation effect is sometimes not ideal. Li Minjia et al. [15] proposed a maximization algorithm (SHDD) based on structural hole and degree discount, which integrated the structural hole idea and centrality idea into the influence maximization problem. J Zhao et al. [16] discussed the identification of influential nodes in unweighted networks, and proposed a measure of node importance, which not only considers its own node importance, but also the influence of other connected nodes. The algorithm conducts propagation experiments on the SI model and obtains a similar effect to the Closeness Centrality algorithm. Han Zhongming et al. [17] proposed an effective node influence measurement model (local triangle centrality—LTC) based on the triangle structure between nodes and neighboring nodes, and carried out experiments on several real, complex networks, indicating that the LTC algorithm can measure the propagation influence of nodes more accurately. E Yu et al. [18] believe that the mutual influence between nodes is the key factor for information dissemination. By reordering existing methods, a new algorithm named RINF is proposed. The algorithm was evaluated using the SIR model. Experimental results show that the RINF algorithm identifies influential nodes in a social network with lower time complexity compared to the benchmark methods. Lv Zhiwei et al. [19] analyzed the shortcomings and limitations of existing methods for identifying influential nodes based on local and global features. They propose an average shortest path centrality metric to identify influential nodes across the network. This metric takes into account the relative change in the average shortest path across the network after each node is removed. To evaluate the performance, the SIR model was used to simulate the performance of the method. The experimental results demonstrate that the proposed method has good performance.

Although the methods mentioned above can better identify influential nodes in social networks, they are all aimed at static social networks and cannot reflect the dynamic and time-sensitive characteristics of social networks. In order to better analyze the rules and characteristics of information transmission in dynamic social networks, some researchers improved the influence maximization algorithm of static social networks and put forward a transmission model and related algorithms suitable for dynamic social networks.

According to the dynamic characteristics of social networks, Tong et al. [20] dispersed the time constraint to each step of seed selection and achieved the goal of maximizing influence under the given time constraint by making each step of seed selection subject to budget constraint. Liu Bo et al [21] illustrated that the time-constrained influence maximization problem is NP-hard and proved the monotonicity and submodularity of the time-constrained influence propagation function. On this basis, they proposed two heuristics based on influence diffusion paths. The validity of the methods is verified on four real data sets. Wei Chen [22] et al. conducted a study around the property that the social network influence maximization problem has a delayed diffusion process and extended the independent cascade model and the linear threshold model to incorporate the time delay of influence diffusion between nodes in social networks. It is proven that the time-critical influence maximization under the extended IC and LT models maintains the submodular properties. To overcome the inefficiency of greedy algorithms, two heuristic algorithms are designed to solve the dynamic social network influence maximization problem with time delays. Yunfang Chen et al. [23] analyzed the problem that the traditional independent cascade model is only adapted to static networks and has a fixed activation probability between nodes. A dynamic social network influence diffusion model with a decay factor is proposed to calculate the activation probability between nodes based on affinity propagation. In order to apply to dynamic social networks, the social network is dynamically sliced according to time slices, so that the activation probabilities can be effectively correlated in different time slices. The experimental results show that the seed node in the model has more chances to activate its neighbor nodes, which can more accurately represent the influence spreading process. Wu Anbiao et al. [24] proposed a propagation model based on the temporal graph by improving the independent cascade model. The propagation probability between nodes is calculated by combining the number of connections between nodes and the PageRank algorithm. Based on this, a two-stage calculation method was proposed to determine the nodes with the greatest influence by optimizing the node marginal effects. Chen Jing et al. [25] proposed an IWCM model applicable to temporal social networks based on temporal graphs for the temporal relationships existing in nodes in dynamic social networks, and based on this, proposed an algorithm including a temporal heuristic phase and a temporal greedy phase to solve for nodes that maximize influence. The method combines the advantages of a heuristic algorithm and greedy algorithm and substantially improves the operation efficiency of the algorithm, but the propagation probability of information between nodes in social networks by the IWCW model only relies on the number of contacts between nodes and cannot accurately measure the influence relationship between nodes in social networks.

In order to better match the propagation characteristics of influence in dynamic social networks, this paper improves the traditional IC model, resets the activation probability among nodes, and constructs a two-stage algorithm to find the set of seed nodes in dynamic social networks according to the ideas in the literature [24,25] to improve the efficiency of solving the influence maximization problem.

## 3. Problem Definition

This section analyzes the working principle of the independent cascade model and explains the characteristics of the model and defines the related concepts of influence maximization in dynamic social networks. In order to explain the problem conveniently, the symbols and their meanings involved in the paper are given in Table 1.

**Figure 1:**
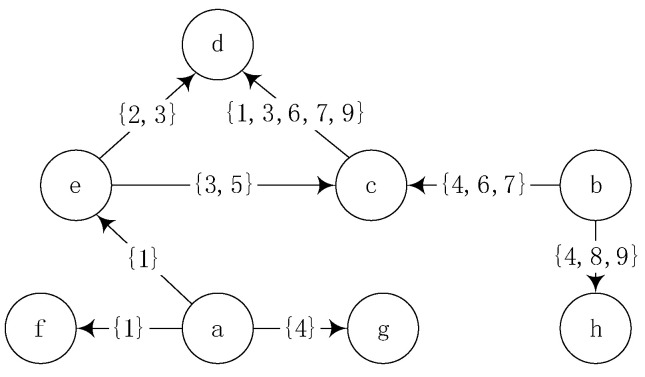
Effective connection diagram of eight nodes.

In $G^{T}\left(V,E,T_{E}\right)$, V denotes the set of nodes in the social network, E denotes the set of connected edges between nodes, and $T_{E}$ represents the set of connected moments between nodes in the network. In a dynamic social network, interaction between nodes can only occur at a given time of contact. For example, in Figure 1, $t\left(e,c\right)=\left\{3,5\right\}$ between node e and node c, it means that node e will contact node c only at time three and time five, and there will be no interactive operation in the rest of the moments although there is a topological connection relationship.

### 3.1. Basic Definitions

**Definition** **1.** Influence maximization in dynamic social networks. In a given dynamic social network, a node set of size k ($S^{*}\subset V$) is found through the corresponding propagation model to maximize the influence range of the set $S^{*}$. This problem can be expressed by Equation (1), and $\sigma_{T}\left(S\right)$ shows the scope of influence of seed set S.

(1) $$S^{*}={\mathrm{argmax}}_{\left|S\right|=K,S\subset V}\sigma_{T}\left(S\right)$$

**Definition** **2.** *Node similarity. An evaluation index that can measure the degree of similarity between adjacent nodes. In this paper, the Jaccard similarity coefficient is used to define the similarity of node u and node v, and its form is shown in Equation (2).*

(2) $$D_{u,v}=\frac{\left|\Gamma_{u}^{\mathrm{in}}\cap \Gamma_{v}^{\mathrm{in}}\right|}{\left|\Gamma_{u}^{\mathrm{in}}\cup \Gamma_{v}^{\mathrm{in}}\right|}$$

*where, $\Gamma_{u}^{in}$ and $\Gamma_{v}^{in}$ represent the in-degree of nodes u and v, respectively, and the similarity has a strong correlation with the trust degree between nodes.*

**Definition** **3.** *Marginal gain. For set functions gain: $2^{V}\to \mathbb{R}$, a subset S⊆V, and an element e∈V in the set, not $\mathrm{gain}\left(u\right)=\mathrm{Spread}\left(S\cup \left\{e\right\}\right)-\mathrm{Spread}\left(S\right)$ as the marginal gain resulting from adding element e to set S.*

**Definition** **4.** *Effective connections. We stipulate that the connection between nodes in a social network is not maintained constant, and only at a particular point in time can the connection between neighboring nodes be realized. It includes the effective ties of edges and effective ties of nodes.*

The effective ties of edges represent the set of time points at which actual interactions occur between any nodes, denoted by $t\left(u,v\right)=\left|\left\{t_{1},t_{2},\cdots t_{n}\right\}\right|$, and $t_{i}$ denotes the time points at which interactions occur between nodes. The effective ties of nodes denote the sum of the effective connections between the target node and its direct neighbors, denoted by $T_{u}$, and the relationship between them is shown in Equation (3).

(3) $$T_{u}=\underset{v\in \Gamma_{u}^{\mathrm{out}}}{\sum }t\left(u,v\right)$$

$\Gamma_{u}^{\mathrm{out}}$ denotes the out-degree neighbor of node u. The efficient linkage satisfies the characteristics of discrete and independent. Whether any node u can activate its neighbor node v must satisfy two conditions. First, the activation operation on the adjacent node v can be effective only when the node u is in the active state. Secondly, the activation process of node u to node v must consider the sequence of time points contained in the valid connection of edges. For nodes $x\in \Gamma_{u}^{\mathrm{in}}$ and $v\in \Gamma_{u}^{\mathrm{out}}$, node v can be activated only when the case of $\forall t_{i}\in t\left(x,u\right)$, $\forall t_{j}\in t\left(u,v\right)$ and $\exists t_{i}<t_{j}$ is satisfied.

**Definition** **5.** *Impact probability. An evaluation index that measures the degree of interaction between adjacent nodes, the commonly used setting method is to set a random number between zero and one independently for the edge connection between any nodes in the social network, or directly take the reciprocal of the number of the edge connection between affected nodes as the standard to evaluate the influence ability of nodes.*

In social networks, the importance of a node can be measured based on the number of directly adjacent users of the node, but there are differences in the influence generated by out-degree neighbors and in-degree neighbors [26]. In order to better reflect the influence of the topology structure and interaction between nodes in the social network on users, this paper refines the influence of out-degree neighbors and in-degree neighbors of nodes on influence propagation according to the method of [27], and introduces scale factor x and y to generate the direct adjacency ${\mathrm{dk}}_{u}$ of the node in the form shown in Equation (4).

(4) $${\mathrm{dk}}_{u}={\mathrm{xk}}_{u}^{\mathrm{in}}+{\mathrm{yk}}_{u}^{\mathrm{out}}$$

$k_{u}^{\mathrm{in}}$ and $k_{u}^{\mathrm{out}}$ represent the number of in-degree neighbors and out-degree neighbors of node u respectively. The scale factor satisfies x+y=1.

**Figure 2:**
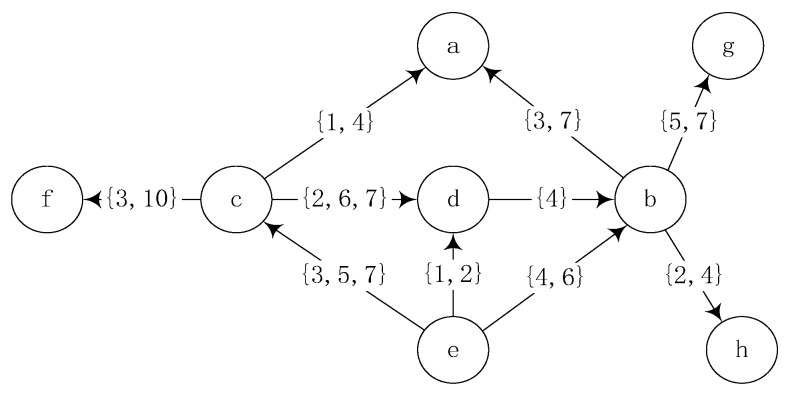
Example network.

Since the influence of nodes in the process of information diffusion will gradually decay with the propagation process, the influence is mainly concentrated in the two-hop range [28,29,30], in order to further accurately assess the influence ability of nodes, we extend the direct adjacency of nodes to the two-hop range to form the indirect adjacency ${\mathrm{Ik}}_{u}$, whose form is shown in Equation (5).

(5) $${\mathrm{Ik}}_{u}=x\sum_{v\in \Gamma_{u}^{\mathrm{in}}}{\mathrm{dk}}_{v}+y\sum_{v\in \Gamma_{u}^{\mathrm{out}}}{\mathrm{dk}}_{v}$$

where, $\Gamma_{u}^{\mathrm{in}}$ and $\Gamma_{u}^{\mathrm{out}}$ represent the neighbor set of in-degree and out-degree of node u, respectively. According to the literature [27], the scale factor x=0.75 is set in this paper. In Figure 2, ${\mathrm{dk}}_{d}={\mathrm{xk}}_{d}^{\mathrm{in}}+{\mathrm{yk}}_{d}^{\mathrm{out}}=2x+y=1.75$. Similarly, ${\mathrm{dk}}_{e}=0.75$, ${\mathrm{dk}}_{a}=1.5$, ${\mathrm{dk}}_{g}=0.75$, ${\mathrm{dk}}_{h}=0.75$. Indirect neighbor degree, ${\mathrm{Ik}}_{b}=x\left({\mathrm{dk}}_{d}+{\mathrm{dk}}_{e}\right)+y\left({\mathrm{dk}}_{a}+{\mathrm{dk}}_{g}+{\mathrm{dk}}_{h}\right)=x\left(1.75+0.75\right)+y\left(1.5+0.75+0.75\right)=2.625$.

The interaction between nodes is carried out in the local topology scope of the target node, we use the importance proportion of the target node in its adjacent nodes to measure the degree of mutual influence between nodes, thus introducing the indirect adjacency degree, which represents the sum of the importance of all adjacent nodes of a node. Therefore, we define the influence probability between nodes as Equation (6).

(6) $$p_{u,v}={\mathrm{dk}}_{u}/{\mathrm{Ik}}_{v},\;v\in \Gamma_{u}$$

For example, in Figure 2, the influence probability of node d on node b is $p_{\mathrm{db}}={\mathrm{dk}}_{d}/{\mathrm{Ik}}_{b}\approx 0.67$. In static social networks, connections between nodes are considered to be constant based on topological relations, which is inconsistent with the dynamic characteristics of actual social networks, the actual situation is that the nodes may be connected several times in a certain period of time, and the process is discrete and independent.

**Definition** **6.** Propagation probability in dynamic social networks. The influence probability between any node pairs in dynamic social networks is based on the local topological connection relationship formed by nodes, which describes a relationship of mutual influence between nodes in the effective connection process based on time series. Use $p_{u,v}^{*}$, and define the form as Equation (7).

(7) $$p_{u,v}^{*}=\left\{\begin{array}{c} 1-{\left(1-p_{u,v}\right)}^{\theta }\\ s.t.\;\theta \in \left[1,\left|T\left(u,v\right)\right|\right] \end{array}\right.$$

*$p_{u,v}$ represents the influence probability of node u on node v. $\left|T\left(u,v\right)\right|$ represents the number of effective connections between nodes in a dynamic social network. θ indicates that node u tries to activate node v for the θth time, the propagation probability increases with the number of activations.*

### 3.2. Independent Cascade Propagation Model

The propagation model is used to simulate the process of information transmission in the real world, it is generally divided into a linear threshold model (LT), an independent cascade model (IC) [2], an infectious disease model [31], etc. Since the research work in this paper is based on the independent cascade model, the working principle of the independent cascade model is mainly introduced here.

In the independent cascade model, social networks are modeled as directed graphs $G=\left(V,E\right)$, where V represents the node set of users, and E represents the set of directed edges of the link relationship between users. Each edge $\left(u,v\right)\in E$ is assigned a probability of influence $p_{u,v}\in \left[0,1\right]$, if there is no connection between nodes, then p=0.

The independent cascade model classifies the state of each node into active and inactive states, at step t = 0, given node set S (defined as seed set) in an active state. Any node u∈S has only one chance to influence its unactivated neighbor node v with probability p, regardless of whether v can be activated, node u will not attempt to activate this neighbor node in the subsequent process. If node u successfully activates neighbor node v at time t, node v becomes active at time t + 1, and it will continue to try to activate other neighboring nodes in an inactive state. If multiple neighbors of v are activated at time t at the same time, the order in which they try to activate v is arbitrary, and the activation process between each pair of nodes is independent of each other. The process is repeated for each active node until there are no active nodes in the network.

## 4. Model and Algorithm

### 4.1. Propagation Model Based on Effective Link

In the independent cascade with effective link (ICEL) model, the network is modeled as $G^{T}\left(V,E,T_{E}\right)$, where $T_{E}$ represents the set of time when there is connection between nodes in the network. In this model, each edge $\left(u,v\right)\in E$ is assigned an influence probability $p_{u,v}^{*}$, which reprsents the influence of node u on node v in a dynamic social network. This value is related to the number of interactions between nodes, and indicates that if two nodes interact frequently, information is more likely to be transmitted between them [32], which means that the influence process between nodes may occur multiple times.

Just like in the IC, the seed set S is set to an active state during the initial stage of propagation. In the step t≥1, any node u∈S can independently activate its inactive neighbor node v with probability $p_{u,v}^{*}$ on the premise of satisfying the effective relation between neighboring nodes (input-degree neighbor and output-degree neighbor). If the activation process is completed at the first time point in the edge efficient linkage, node v will change to the active state at step t and continues to propagate the influence at step t + 1. If u fails to activate node v successfully for the first time, node u will activate v again in the subsequent process according to the similarity between nodes until node v is activated or all the time points in the edge effective connection are exhausted. The diffusion process stops when all active nodes have activated their neighbors and no new nodes are activated.

It should be noted that the ICEL model degenerates into an independent cascade model when the effective connection between any nodes in a social network is maintained continuously. In the original independent cascade model, it is believed that as long as there is a connection relationship between nodes, the activation process will occur. However, the limited activation is constituted as one time. Therefore, if we only consider the overall impact in operation, there is no need to introduce a valid linkage. However, if the sequential relation and effective relation between nodes are considered, satisfying the effective connection is an important factor to determine the seed set.

In this paper, the similarity between nodes is used to measure the change of activation probability with activation times when node u tries to activate node v several times. Given a similarity threshold D, if the similarity between nodes u and v is $D_{u,v}>D$, it is considered that the activation probability increases with the activation times. $D_{u,v}=D$ is considered the activation probability that does not change with the number of activations. $D_{u,v}<D$ is considered the activation probability that decreases with the increase in activation times.

When node u attempts to activate node v, node u first deactivates node v with probability $p_{u,v}^{*}=1-{\left(1-p_{u,v}\right)}^{1}$, if node v is not activated successfully for the first time, and there is still a moment when u contacts v, then the similarity is judged. If the similarity is less than or equal to the given threshold D, it is impossible for node u to activate node v thereafter. Therefore, node u will no longer try to activate node v at subsequent contact moments. If the similarity is greater than the given threshold D, node u deactivates node v with probability $p_{u,v}^{*}=1-{\left(1-p_{u,v}\right)}^{2}$ again, and the process continues until there is no longer an effective connection between node u and node v.

As shown in Figure 2, when node c attempts to deactivate node d, the earliest time when node c itself may be activated is considered first. The minimum contact time between node c and its in-degree neighbor node e is three, so it is said that node c may be activated by node e at time three at the earliest, so the only valid contact moments between node c and node d are moments six and seven. Node c deactivates node d at time six with the propagation probability $p_{c,d}^{*}=1-{\left(1-p_{c,d}\right)}^{1}=0.7059$, if node d is not activated, judge whether node c is similar to node d. If not, node c will not try to activate node d at moment seven. Otherwise, node c deactivates node d again at time seven with the propagation probability $p_{c,d}^{*}=1-{\left(1-p_{c,d}\right)}^{2}=0.9135$. The execution process of the ICEL model is shown in Algorithm 1.
**Algorithm 1.** The work principle of ICEL model**Input:** Activation threshold β, Node u and Node v, Similarity threshold D
**Output:** If node u activates node v, return true; otherwise, return false(1)  Calculate the probability $p_{u,v}^{*}=1-{\left(1-p_{u,v}\right)}^{1}$ that node u activates v(2)  if $p_{u,v}^{*}\geq \beta$
(3)   return true(4)  Calculate $D_{u,v}$ according to Equation (2)(5)  if $D_{u,v}>D$
(6)   for θ=2 to $\left|T\left(u,v\right)\right|$ do(7)    $p_{u,v}^{*}=1-{\left(1-p_{u,v}\right)}^{\theta }$
(8)    if $p_{u,v}^{*}\geq \beta$
(9)     return true(10) return false

Step (1) calculate the probability that node u activates node v for the first time. Step (2) to Step (3) indicates that node u activates node v for the first time and the activation succeeds; Step (4) calculates the similarity between the two nodes. Step (5) to Step (9) indicates that node u fails to activate node v for the first time, and the similarity between the two nodes needs to be judged, if the similarity is greater than the similarity threshold D, node u deactivates node v with a certain probability again. Step (10) indicates that node u fails to activate node v after multiple attempts.

### 4.2. Characteristics of the ICEL Model

Because the ICEL model is a special case of the independent cascade model, the problem of maximizing influence with the ICEL model also belongs to the NP-hard problem [2]. In this part, we introduce the properties of the $\sigma_{T}\left(S\right)$ function of the problem of maximizing influence using the ICEL model in dynamic social networks, and prove that the approximate optimal solution of 1-1/e-ε can be obtained by using a greedy algorithm to solve this kind of problem.

**Theorem** **1.** *The influence function $\sigma_{T}\left(S\right)$ in the ICEL model satisfies monotonicity and sub-model.*

**Proof.**  In the ICEL model, each social network can be regarded as a random graph. Each edge $\left(u,v\right)\in E$ in the figure is endowed with the influence probability $p_{u,v}$ formed by the topological relationship between nodes. Here, $p_{u,v}$ controls the possibility that node u activates v, each node u∈V has a distributed $T_{u}$ with effective connection with its neighbors and controls the number of interactions between node u and its neighbors. Let X represent all probability spaces composed of all influence propagation graphs. By flipping a coin for each edge $\left(u,v\right)\in E$, it is determined whether the edge is affected by the influence probability $p_{u,v}$ and the effective connection distribution $T_{u}$, which exists in the influence propagation diagram. Thus, we construct $\sigma_{T}\left(S\right)=\sum_{x\in \chi }p\left(x\right)\sigma_{T,x}\left(S\right)$, where $p\left(x\right)$ represents the probability of determining the propagation graph x, and $\sigma_{T,x}\left(S\right)$ represents that in the determined propagation graph x, set S can affect the number of nodes through effective connections.

For any deterministic impact graph x∈X, $\sigma_{T,x}\left(S\right)$ is equivalent to starting from set S, and the length of at least one path formed through effective connection is less than the number of impact nodes that can be reached on the path of η∈ℝ. $\sigma_{T,x}\left(S\right)\leq \sigma_{T,x}\left(S\cup \left\{u\right\}\right)$ is easy to find, so $\sigma_{T,x}\left(S\right)$ is monotonic. Because of $\sigma_{T}\left(S\right)=\sum_{x\in \chi }p\left(x\right)\sigma_{T,x}\left(S\right)$ and $p\left(x\right)\in \left(0,1\right]$, $\sigma_{T}\left(S\right)$ satisfies monotonicity, which is proven.

Let S⊆T⊆V, any node u∈V. We first consider determining the graph x∈X. $\sigma_{T,x}\left(S\cup \left\{u\right\}\right)-\sigma_{T,x}\left(S\right)$ refers to the number of nodes included in the impact subgraph starting from node u and the path length is η, rather than starting from set S, the impact path length is the number of η impact nodes. $\sigma_{T,x}\left(S\cup \left\{u\right\}\right)-\sigma_{T,x}\left(S\right)\geq \sigma_{T,x}\left(T\cup \left\{u\right\}\right)-\sigma_{T,x}\left(T\right)$ exists because of S⊆T. Therefore, $\sigma_{T,x}\left(S\right)$ is submodular, especially $\sigma_{T}\left(S\right)$ is a nonnegative linear combination of submodular function $\sigma_{T,x}\left(S\right)$, so $\sigma_{T}\left(S\right)$ is submodular, and the proof is completed. □

### 4.3. OEL Algorithm

Degree centrality is one of the most common heuristic methods. It measures the importance of nodes from the perspective of local topology according to the number of directly adjacent nodes. In a directed graph, degree centrality refers specifically to the out-degree neighbors of nodes. The higher the number of out-degree neighbors, the more important the nodes are in the process of information diffusion. However, in dynamic social networks, the connections between nodes does not play a role all the time, and the transmission of information is accomplished only through a limited connection. Therefore, degree centrality cannot be used to measure the influence ability of nodes in dynamic social networks. For example, in the directed network shown in Figure 1, node a has three out-degree neighbor nodes and has one effective connection with each node. Node b has two out-degree neighbor nodes and can make three effective connections with each out-degree neighbor. Only from the perspective of degree centrality, does node a play a greater role in information dissemination. However, the connection process between nodes does not remain unchanged. Although there is a connection relationship on the network topology, the number of effective connections is small. Only at t = 2, there is an incentive effect on adjacent nodes, while at other times, there is no impact on adjacent nodes, and the information cannot be effectively diffused. It can be seen that the effective connection between nodes can effectively promote the dissemination of information in social networks. On the other hand, the influence ability of a node cannot only consider the effective connection. In Figure 1, node c has only one out-degree neighbor node d, and the effective connection between it and node d is $T_{c}=t\left(c,d\right)=5$. The out-degree neighbors of node a are e, f, g, and the effective connection times of this node are $T_{a}=t\left(a,e\right)+t\left(a,f\right)+t\left(a,g\right)=3$. It can be seen from the figure that $T_{c}>T_{a}$ at this time. Since the out-degree degree of node d is 0, node c can affect at most one node, while node a has the potential ability to influence at least three nodes. It can be seen that the above methods cannot accurately measure the influence of nodes in dynamic social networks.

In this paper, we build a dynamic social network node influence evaluation algorithm named outdegree effective link (OEL) by integrating the two factors of degree centrality and effective connection. The algorithm includes the heuristic stage and the greedy stage. In the heuristic stage, we screen the top $\alpha k\left(\alpha k\geq k\right)$ nodes with the largest $\mathrm{OEL}\left(\cdot \right)$ according to Formula (8), to form a set of potential influence nodes. α is the regulation factor, and the value is empirical. It is necessary to determine the value of α according to the results of the OEL algorithm running on the dataset.

(8) $$\mathrm{OEL}\left(u\right)=\gamma \times \left|\Gamma_{u}^{\mathrm{out}}\right|+\left(1-\gamma \right)\times T_{u}$$

where, $\left|\Gamma_{u}^{\mathrm{out}}\right|$ denotes the outdegree of node u, which represents the inherent attribute of the node, and is the upper limit of the local influence of the node. $T_{u}$ is the effective connection between nodes of u, which indicates the degree of effort that the node tries to influence other nodes and determines whether the node influence can reach the upper limit. γ is a tuning coefficient used to adjust the proportion of two types of attributes in node influence. αk and k represent the size of potential influence set and seed set respectively.

In the greedy stage, we look for the node that can produce the greatest influence in the potential influence set, apply the greedy algorithm to calculate the influence range gain of each node in the set in turn, and select the node with the largest gain to be selected into the seed set until the number of selected seed nodes reaches k. We use submodularity to screen the seed nodes in the greedy phase [4], which drastically reduces the problem of low running efficiency caused by the greedy Algorithm 2.
**Algorithm 2.** OEL algorithm**Input:**$G_{T}$(V,E,$T_{E}$), Seed size k, Threshold of activation β, Alternative seed set $S_{1}$, Regulatory factors α**Output:** Seed set S(1) Initialize S=∅, $S_{1}=\emptyset$
(2) For i = 1 to αk do(3)  $v=\max\left\{\mathrm{Oct}\left(u\right)|u\in V\backslash S_{1}\right\}$;(4)  $S_{1}=S_{1}\cup v$;(5) Endfor(6) For u in $S_{1}$ do(7)  $\mathrm{gain}\left(u\right)=\mathrm{Spread}\left(S\cup \left\{u\right\}\right)-\mathrm{Spread}\left(S\right)$;(8) Endfor(9) Sorted $S_{1}$ according $\mathrm{gain}\left(\cdot \right)$ in descending(10) $x=S_{1}.\mathrm{pop}\left(0\right)$;(11) S=S∪x;(12) For i = 2 to k do(13)   gain($S_{1}\left[0\right]$) = Spread(S$\cup \left\{S_{1}\left[0\right]\right\})-\mathrm{Spread}\left(S\right);$
(14)   if(gain($S_{1}\left[0\right]$) > gain($S_{1}\left[1\right]$))(15)    $x=S_{1}.\mathrm{pop}\left(0\right)$;(16)    S=S∪x;(17)   else(18)    For u in $S_{1}$ do(19)     $\mathrm{gain}\left(u\right)=\mathrm{Spread}\left(S\cup \left\{u\right\}\right)-\mathrm{Spread}\left(S\right)$;(20)    Endfor(21)    Sorted $S_{1}$ according $\mathrm{gain}\left(\cdot \right)$ in descending;(22)    $x=S_{1}.\mathrm{pop}\left(0\right)$;(23)    S=S∪x;(24)   Endif(25) Endfor(26) Return S

During the execution of the OEL algorithm, Step (1) indicates that the initialization seed set s and the candidate seed set $S_{1}$ are empty sets. Step (2) to Step (5) take the OEL value as the standard to measure the importance of nodes and filter the top αk nodes to build an alternative seed set. Step (6) to step (11) means that the marginal benefits of each node in the alternative seed set are calculated and sorted in turn, and the node with the largest marginal benefits is added to the seed set; Step (12) to Step (25) use the k-1 seed nodes after sub model selection. Finally, the seed set s is returned.

During the operation of the algorithm, the nodes in the candidate seed set always maintain the descending order of influence gain. $x=S_{1}.\mathrm{pop}\left(0\right)$ indicates that the node with the maximum marginal gain is ejected from the candidate seed set.

### 4.4. Time Complexity Analysis

We analyze the running time of the OEL algorithm. In the heuristic phase of the algorithm, we need to calculate OEL values for all nodes in turn. Since OEL values are only related to the out-degree of nodes and the effective connection of nodes, their time complexity is constant, represented by $O\left(N\bar{C}\right)$, $\bar{C}$ represents the average effective connection of nodes and N represents the number of nodes. At this stage, αk candidate nodes should be selected from the whole social network as candidate seed sets. The time complexity is $O\left(\alpha k*N\right)$ so the total time complexity of the heuristic stage is $O\left(N\bar{C}\right)+O\left(\alpha k*N\right)$. Step (6)~step (9) calculate the gain of each node in the alternative seed set and arrange it in descending order. The time complexity is $O\left(\mathrm{klogk}\right)$. Step (12)–step (25) realize the screening of seed nodes in the greedy stage. Compared with the greedy algorithm, it is not necessary to calculate the impact gain of all nodes in the social network in turn, reduce the investigation space to the alternative seed set, and further shorten the running time of the algorithm in combination with the secondary model. The time complexity of this part is $O\left({\alpha k}^{2}\bar{C}\right)$. Therefore, the total time complexity of the algorithm is $O\left(N\bar{C}\right)+O\left(\alpha k*N\right)+O\left(\mathrm{klogk}\right)+O\left({\alpha k}^{2}\bar{C}\right)$.

## 5. Experiment and Evaluation

All experiments in this paper are run on the same PC with the following configuration: Intel(R) Core(TM), 1.60 GHz, memory 8.00 GB, Windows10 operating system. All the codes involved in the experiment were implemented in Python language on PyCharm.

### 5.1. Experimental Data

In order to verify the effectiveness of the method, we carried out experiments on four real data sets of different sizes, which were dolphins, ca-netscience, netscience and p2p-Gnutella08. The four data sets all came from http://networkrepository.com on 15 February 2009. Among them, dolphins were made by Lusseau and others in New Zealand, to observe the living habits of 62 wide kiss dolphins for a long time. They found that the interaction of these dolphin showed a specific mode, and they also built a social network with 62 node. ca-netscience which was known as the network of scientists. When an article was finished by several scientists together, they formed a connection. Netscience was a network created by both network theory and experimental scientists. p2p-Gnutella08 was a scientific cooperation between the scientific research articles on the category of general principles of gravity and the category of electronic universe. If the author i and the author j jointly write a thesis, the drawings would cover the edges from i to j. If the thesis was jointly written by author k, a completely connected sub picture would be produced on k node. The experimental data set was as shown in Table 2. In order to reflect the changes of the dynamic social network, we distributed the random number of effective contacts to all the connections of a given social network within the range of [1,10] to simulate the effective connections between users.

In the dataset shown in Table 2, M denotes the number of nodes, N denotes the number of edges, MaxD denotes the maximum degree of a node, MinD denotes the minimum degree of a node, ⟨D⟩ denotes the average degree values of a node, and T denotes the number of triangles formed between nodes.

### 5.2. Experimental Settings

In order to verify the experimental effectiveness of the OEL algorithm on dynamic social networks, random, degree, degreediscount, betweeness, and greedy are selected as comparison algorithms in this paper. The random algorithm (random) is a method of randomly selecting nodes in the network, which means that the set of seed nodes with the greatest influence is randomly selected without any strategy. This method is usually used as a benchmark comparison algorithm, formally as shown in Equation (9).

(9) $${\mathrm{RC}}_{i}=\mathrm{Random}\left(G,i\right)$$

The degree centrality algorithm (degree) [12] evaluates the influence ability of node according to the number of direct adjacent nodes of node I, which is formally expressed as Equation (10).

(10) $${\mathrm{DC}}_{i}=\left(\begin{array}{c} N\\ 2 \end{array}\right)\underset{j\in G\backslash i}{\sum }A_{i,j}$$

The j represents any node in graph G except node i. $A_{i,j}=1$ when there is a connecting edge between node i and node j; otherwise, $A_{i,j}=0$. The degree centrality algorithm is based on local topological structure. The greater the degree of nodes, the more potential influences of their direct neighboring nodes.

The degreediscount algorithm [33] is a variant of the degree centrality algorithm. The algorithm iteratively selects the optimal node based on degree centrality. After each round of selection, the degreediscount operation is performed on the direct adjacent nodes of the optimal node, formally expressed as Equation (11).

(11) $${\mathrm{DDC}}_{i}=\mathrm{argmax}\left({\mathrm{DC}}_{i}-2S_{i}-\left({\mathrm{DC}}_{i}-S_{i}\right)S_{i}{}^{\beta }\right)$$

$S_{i}$ represents the set of activated nodes in the direct adjacent nodes of the current node i, and β represents the activation probability between adjacent nodes. This algorithm can effectively alleviate the evaluation error caused by influence overlap.

The betweenness algorithm [13] evaluates the influence of nodes according to the number of shortest paths of a node in the network, which can be expressed as Equation (12).

(12) $${\mathrm{BC}}_{i}=\left(\begin{array}{c} N\\ 2 \end{array}\right)\sum_{u\neq i\neq v}\frac{\left|{\mathrm{path}}_{\mathrm{uv}}\left(i\right)\right|}{\left|{\mathrm{path}}_{\mathrm{uv}}\right|}$$

$\left|{\mathrm{path}}_{\mathrm{uv}}\right|$ is the number of shortest paths from node u to node v, $\left|{\mathrm{path}}_{\mathrm{st}}\left(i\right)\right|$ is the number of shortest paths through node i, and N is the number of nodes in the network.

The greedy algorithm [2] requires that the marginal influence of all inactive nodes be calculated every time, and the node with the maximum gain of influence is selected into the seed node set, which is formally expressed as Equation (13).

(13) $${\mathrm{KK}}_{i}=\mathrm{argmax}\left(\left|I\left(S_{i}\cup \left\{u\right\}\right)\right|-\left|I\left(S_{i}\right)\right|\right)$$

$I\left(S_{i}\right)$ represents the influence of the current set $S_{i}$, and $I\left(S_{i}\cup \left\{u\right\}\right)$ represents the influence after any node u joins the set $S_{i}$.

In the proposed algorithm OEL, the tuning coefficient γ is set as 0.6, the similarity threshold D is set as 0.5, and the Scale factor x is set as 0.75. In addition, in order to make the experimental comparison effect more obvious, the inter-node activation threshold values of the four data sets were set as 0.5, 0.1, 0.1 and 0.5 respectively after several tests.

### 5.3. Experiment and Result Analysis

#### 5.3.1. Parameter Analysis of the OEL Algorithm

The OEL algorithm is divided into two phases, heuristic and greedy. The number of nodes selected in the heuristic phase affects the quality of the seed nodes selected in the subsequent greedy. If the number of nodes selected in the heuristic phase is too small, it will lead to poor propagation of the final selected nodes, while too many selected nodes will reduce the operation efficiency of the algorithm. In order to determine the proportional relationship between the nodes selected in the heuristic stage and the greedy stage, we conducted comparative experiments on different regulatory factors to verify the reasonable value range of α.

**Figure 3:**
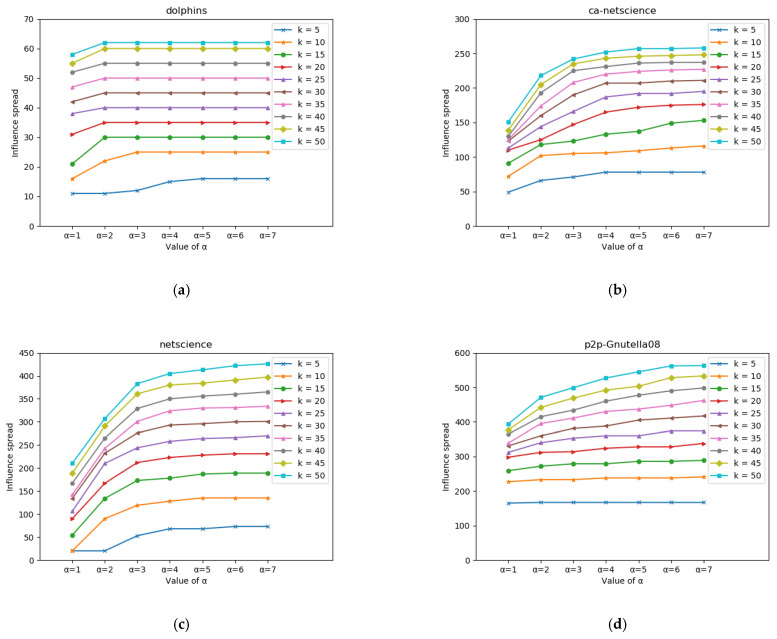
Influence of regulatory factors on transmission range. (**a**) The influence of regulatory factor α on transmission range on dolphins dataset. (**b**) The influence of regulatory factor α on transmission range on ca-netscience dataset. (**c**) The influence of regulatory factor α on transmission range on netscience dataset. (**d**) The influence of regulatory factor α on transmission range on p2p-Gnutella08 dataset.

In the experimental process, the number of seed nodes k was restricted to be distributed in the range of 5~50 on the four data sets, and the value space of α was set as 1~7. The influence of regulatory factor α on the propagation range is shown in Figure 3. It can be seen from the experimental results that on the dolphins dataset, due to the small size of the network, the regulatory factors have little influence on the transmission range, especially when α>3, the change of influence tends to be stable. In the ca-NetScience, NetScience and P2P-Gnutella08 data sets, the range of influence increased with the increase in seed set size, and gradually stabilized after k = 40. Especially in ca-Netscience and P2P-Gnutella08 datasets. However, it can be seen from the figure that when α>4, influence changes without the set of scale seeds on each data set are relatively stable. In order to balance the time efficiency of the algorithm and the spread range of influence, it is more practical to determine α=4, and subsequent experiments are also carried out under this condition.

#### 5.3.2. Scope of Transmission

In this section, we compare the propagation range of the proposed algorithm OEL with five typical algorithms. The experiment selects 5 to 50 seed nodes (step size 5) to simulate propagation.

**Figure 4:**
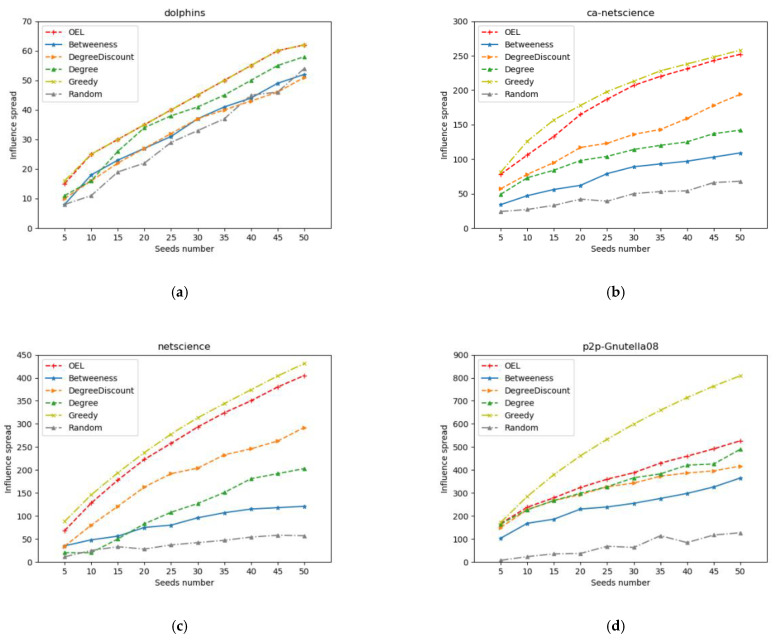
Comparison of influence areas. (**a**) Propagation range of each type of algorithm on dolphins dataset. (**b**) Propagation range of each type of algorithm on ca-netscience dataset. (**c**) Propagation range of each type of algorithm on netscience dataset. (**d**) Propagation range of each type of algorithm on p2p-Gnutella08 dataset.

Figure 4 shows the comparison of the propagation influence of the six algorithms on the four datasets, the horizontal coordinate is the number of seed nodes and the vertical coordinate is the propagation range of the seed nodes. It can be seen that the greedy algorithm achieves the best effect in all data sets, followed by the OEL algorithm, and the random algorithm has the worst effect. In the dolphins data set, the OEL algorithm proposed in this paper achieves the same effect as the greedy algorithm except that the number of seed nodes is five. In the ca-netscience and netscience datasets, our algorithm achieves close propagation effects to greedy. In the p2p-Gnutella08 dataset, although our algorithm is quite different from greedy, it is still better than other algorithms except greedy.

#### 5.3.3. Time Comparison

In order to further verify the effectiveness of the OEL algorithm, we compared the time efficiency of each algorithm in selecting different numbers of seed nodes. Since the Random algorithm has a high time efficiency in seed node excavation, the running time is not given here. The time efficiency of other algorithms is shown in Table 3.

It can be seen from Table 3 that on the dolphins and ca-netscience datasets, the operating efficiency of the OEL algorithm is weaker than that of the methods (degreediscount algorithm and degree algorithm) that rely on local network features to identify target nodes. However, compared with the greedy algorithm, the OEL algorithm reduces the search space of the influence maximization problem in the heuristic stage, and combined with the submodularity characteristics, the operating efficiency is significantly improved. In the netscience dataset, the running efficiency of the OEL algorithm outperforms greedy and betweeness when k is taken from 5 to 15, and only outperforms greedy after k > 15, which indicates that the weight occupied by the greedy process by the OEL algorithm leads to the increase in running time as the number of selected seed nodes increases. In the p2p-Gnutella08 dataset, the OEL algorithm outperforms greedy and betweeness, indicating that our algorithm is more suitable than betweeness for social networks with larger data sizes.

In conclusion, applying the OEL algorithm to search for seed sets for influence propagation in dynamic social networks can achieve a similar effect to that of greedy algorithm, which is significantly higher than other heuristic algorithms. Compared with the greedy algorithm, the OEL algorithm has a significantly reduced running time and is more suitable for exploiting seed node sets in larger social networks.

## 6. Summary

In this paper, we study the influence maximization problem in dynamic social networks. By improving the classical independent cascade propagation model, we take the local topological relationship between nodes and effective links as the influence probability between nodes in dynamic social networks. In addition, we also propose a two-stage influence maximization algorithm, OEL, based on degree centrality and effective link. The algorithm forms a candidate seed set according to the OEL value in the heuristic stage, which reduces the optimization space of the algorithm. In the greedy stage, the influence gain generated by each node is determined by combining the submodularity characteristics, and the efficiency of the algorithm is improved. Through simulations on real data sets, we find that the proposed OEL algorithm is more effective than other heuristic methods to discover the seed set that maximizes influence in dynamic social networks. Compared with the greedy algorithm, the OEL algorithm has better time efficiency.

In the future, we will carry out further research in the following aspects:

1. On the basic dynamic social networks, in order to avoid the problem of influence overlap, construct new metrics to explore the seed set of influence maximization;
2. Modeling of the influence maximization problem in a time-constrained and cost-constrained dynamic network, so as to better measure the information dissemination process in a dynamic social network.

## Figures and Tables

**Table 1:** Symbol Description.

Notations	Definition
$G^{T}\left(V,E,T_{E}\right)$	Dynamic social network
$p_{u,v}$	Activation probability of node u to v in static network
$p_{u,v}^{*}$	Activation probability of node u to v in dynamic network
$\Gamma_{u}^{\mathrm{out}}$	Out edge neighbor of node u
$\Gamma_{u}^{\mathrm{in}}$	In edge neighbor of node u
$t\left(u,v\right)$	The set of effective contact times between node u and node v
γ	Tuning coefficient
D	Similarity threshold between two nodes
β	Activation threshold
θ	Activation times
αk	Number of alternative seed nodes
k	Number of seed nodes

**Table 2:** Experimental data set parameters.

Data Set	M	N	MaxD	MinD	<D>	T
dolphins	62	159	12	1	5	285
ca-netscience	379	914	34	1	4	2.8 K
netscience	1.6 K	2.7 K	34	0	3	11.3 K
p2p-Gnutella08	6.3 K	20.8 K	97	1	6	7.1 K

**Table 3:** Comparison of algorithm running time.

Data Set	Number of Seeds	Algorithm Running Time (Seconds)				
		OEL	Betweeness	DegreeDiscount	Degree	Greedy
dolphins	k = 5	0.00298	0.00182	0.00032	0.00005	0.03191
	k = 10	0.01894	0.00185	0.00038	0.00009	0.12566
	k = 15	0.03092	0.00186	0.00043	0.00011	0.21542
	k = 20	0.04092	0.00189	0.00044	0.00014	0.29721
	k = 25	0.04787	0.00193	0.00049	0.00015	0.32708
	k = 30	0.05485	0.00197	0.00049	0.00017	0.36907
	k = 35	0.05883	0.00199	0.00051	0.00018	0.42582
	k = 40	0.06383	0.00200	0.00054	0.00020	0.46381
	k = 45	0.07879	0.00202	0.00062	0.00021	0.50266
	k = 50	0.08076	0.00203	0.00064	0.00023	0.53354
ca-netscience	k = 5	0.03990	0.02873	0.00154	0.00034	1.01827
	k = 10	0.18747	0.02881	0.00156	0.00049	3.33109
	k = 15	0.39994	0.02886	0.00165	0.00071	6.94841
	k = 20	0.66926	0.02901	0.00166	0.00087	10.66042
	k = 25	1.10308	0.02989	0.00173	0.00098	15.06065
	k = 30	1.22966	0.03007	0.00179	0.00122	18.93035
	k = 35	2.15124	0.03047	0.00194	0.00134	25.21152
	k = 40	2.99598	0.03089	0.00199	0.00143	29.50102
	k = 45	3.59338	0.03130	0.00202	0.00161	34.58282
	k = 50	4.02626	0.03175	0.00213	0.00186	41.33540
netscience	k = 5	0.04986	0.57858	0.00516	0.00141	6.41057
	k = 10	0.18758	0.57941	0.00544	0.00210	23.41469
	k = 15	0.50365	0.58139	0.00559	0.00272	47.60400
	k = 20	0.90489	0.58219	0.00560	0.00352	73.26150
	k = 25	1.59267	0.58240	0.00572	0.00411	109.89939
	k = 30	2.92622	0.58416	0.00591	0.00480	142.60642
	k = 35	3.62019	0.58432	0.00603	0.00554	194.50416
	k = 40	4.41875	0.58439	0.00615	0.00612	223.39536
	k = 45	5.28926	0.58608	0.00620	0.00674	264.54489
	k = 50	6.62509	0.58975	0.00649	0.00757	320.53333
p2p-Gnutella08	k = 5	0.06084	65.30030	0.03375	0.00722	53.91473
	k = 10	0.12068	66.15388	0.03440	0.01075	207.79511
	k = 15	0.51766	66.58277	0.03485	0.01340	463.83709
	k = 20	0.73904	66.98780	0.03491	0.01636	846.52928
	k = 25	1.00833	66.99675	0.03512	0.01938	1280.93165
	k = 30	1.26561	67.00286	0.03541	0.02290	1745.55211
	k = 35	1.76430	67.02671	0.03574	0.02579	2256.53356
	k = 40	2.01059	67.06866	0.03607	0.02986	2791.33391
	k = 45	2.45837	67.18037	0.03656	0.03130	3371.98843
	k = 50	2.81152	67.38176	0.03780	0.03610	4015.44560
